# A core root bacteria contribute to plant growth and anisodine accumulation of *Anisodus tanguticus*

**DOI:** 10.1186/s12870-023-04690-1

**Published:** 2023-12-19

**Authors:** Bo Wang, Chen Chen, Yuanming Xiao, Kaiyang Chen, Juan Wang, Lingling Wang, Jianan Li, Zongxiu Kang, Guoying Zhou

**Affiliations:** 1 CAS Key Laboratory of Tibetan Medicine Research, Northwest Institute of Plateau Biology, Xining, 810008 China; 2 College of Life Sciences, Huaibei Normal University, Huaibei, China; 3 University of Chinese Academy of Sciences, Beijing, 100049 China; 4 Resource institute for Chinese and Ethnic Materia Medica, Guizhou University of Traditional Chinese Medicine, Guiyang, 550000 China; 5 Qinghai University, Xining, 810016 China; 6 Datong Beichuan Heyuan District National Nature Reserve, Xining, 810100 China

**Keywords:** Tibetan medicines, *Anisodus tanguticus*, Core microbiomes, Secondary metabolites, Tropane alkaloid, Endophytes

## Abstract

**Background:**

Although it is well recognized that core root microorganisms contribute to plant health and productivity, little is known about their role to the accumulation of secondary metabolites. The roots of *Anisodus tanguticus*, a traditional herbal medication utilized by Tibetan medicine, are rich in tropane alkaloids. We collected wild *A. tanguticus* populations throughout a 1500 km transect on the Qinghai-Tibetan Plateau.

**Results:**

Our results showed that despite sampling at a distance of 1500 km, the root of *A. tanguticus* selectively recruits core root bacteria. We obtained 102 root bacterial core OTUs, and although their number only accounted for 2.99% of the total, their relative abundance accounted for 73% of the total. Spearman correlation and random forest analyses revealed that the composition of core root microbiomes was related to anisodine contents, aboveground biomass and nitrogen contents of *Anisodus tanguticus*. Among them, the main role is played by *Rhizobacter*, *Variovorax*, *Polaromonas*, and *Mycobacterium* genus that are significantly enriched in roots. Functional prediction by FAPROTAX showed that nitrogen-cycling microorganisms and pathogenic bacteria are strongly associated with anisodine contents, aboveground biomass and nitrogen contents of *Anisodus tanguticus*.

**Conclusions:**

Our findings show that the root selectively recruits core root bacteria and revealed that the core microbiomes and microbial functions potentially contributed to the anisodine contents, aboveground biomass and nitrogen contents of the plant. This work may increase our understanding of the interactions between microorganisms and plants and improve our ability to manage root microbiota to promote sustainable production of herbal medicines.

**Supplementary Information:**

The online version contains supplementary material available at 10.1186/s12870-023-04690-1.

## Introduction

*Anisodus tanguticus* (Maxim.) Pascher, a perennial herb that belongs to the family Solanaceae, is a traditional herbal medicine used by Tibetan medicine. It is found primarily in the alpine and subalpine belts of the Qinghai-Tibetan Plateau at elevations ranging from 2 200 to 4 200 m. Its roots are rich in anisodamine, atropine, scopolamine and anisodine, making it an essential resource plant for tropane alkaloids extraction [1]. It has the effects of promoting blood circulation and removing blood stasis, analgesic and antispasmodic, and hemostasis, and is commonly used in the treatment of traumatic fractures and bleeding, malignant sores, as well as pain caused by canker, chronic or acute gastroenteritis and biliary ascariasis [2, 3]. The demand for *A. tanguticus* is increasing as industrialization and marketization of Tibetan medicine progress. However, Chen et al. collected *A. tanguticus* samples from the Sichuan, Gansu and Qinghai provinces, and showed significant differences in alkaloid content [4]. The mechanism of *A. tanguticus* quality formation research can help to increase resource utilization efficiency and meet market demand.

Many studies have revealed that secondary metabolite production is closely related to light, rainfall, temperature, and soil, among other factors [5]. However, with the development of high-throughput sequencing technology, more studies have revealed that plant microbiomes, particularly endophytes, might influence secondary metabolite accumulation [6–11]. Endophytes are a type of microbiome that lives in healthy plant tissues but does not cause disease in the host [12]. Endophytes have been shown in numerous studies to boost the accumulation of bioactive chemicals in medicinal plants [13, 14]. Firstly, secondary metabolites can be directly secreted by endophytes. Secondly, endophytes can produce intracellular or extracellular enzymes to transform plant metabolites into new active molecules to enhance the content of secondary metabolites. Finally, endophytes can influence plant metabolic pathways via the elicitor effect or lateral gene transfer to promote the production of active ingredients in medicinal plants [15, 16]. According to research on *Rheum palmatum* and *Cynomorium songaricum* from various sites, positive correlations were found between the diversity and abundance of endophytic fungi and the secondary metabolites, respectively [6, 7]. *A. tanguticus* root-associated microbial communities and their relationships with secondary metabolites, however, have not been studied.

Throughout their long-term evolution, endophytes and their host plants established a mutually beneficial relationship [17, 18]. It has been proposed that plants and their associated microbial communities are collectively forming holobionts and should not be considered a separate entity [19]. Many studies have shown that a subset of the plant microbiota, known as the core microbiota, is reproducibly enriched in plant roots [20, 21]. Despite the fact that the local soil microbial reservoirs vary depending on the environment and location, the core microorganisms have dependable connections to their hosts [22, 23]. The core microbiota is composed of key microbial taxa that contribute to plant growth, nutrient uptake, and resistance to biotic and abiotic stresses [24–26]. According to a recent study, a substantially conserved core bacterial microbiota with nitrogen-fixing capacity occupies the xylem sap of maize plants [27]. *Spartina alterniflora* in salt marshes has a core root microbiome capable of sulfur oxidation and sulfate reduction [28]. Recent research on medicinal plants such as *Glycyrrhiza uralensis* and *Panax notoginseng* has revealed a close relationship between the diversity and composition of core rhizosphere and root microbiomes, and the concentration of plant secondary metabolites [9, 29]. Core root microorganisms may exist in *A. tanguticus* from various locations, however the core root microbiome has not yet been identified.

In this study, we collected wild *A. tanguticus* populations throughout a 1500 km transect on the Qinghai-Tibetan Plateau. The tropane alkaloids content and endophytic bacterial and fungal diversity and composition were analyzed using HPLC and high-throughput sequencing. The objectives of the study were to (i) identify core root microbiomes of *A. tanguticus* at a large spatial scale; (ii) investigate the core microbiomes related to tropane alkaloids, aboveground biomass, and root nitrogen of *A. tanguticus*; (iii) clarify the core microbial functions related to tropane alkaloids, aboveground biomass and root nitrogen of *A. tanguticus*. We hypothesise that a core microbiome exists in the roots of *A. tanguticus* and their composition and function were associated with tropane alkaloids, aboveground biomass and root nitrogen of *A. tanguticus*.

## Materials and methods

### Sample collection

We collected natural *A. tanguticus* populations at 58 sites across 1500 km of the Qinghai-Tibetan Plateau, with a latitudinal range from 28°50′ N to 37°54′ N and a longitudinal range from 100°17′E to 99°35′E (Fig. S1). The transect’s mean annual temperature (MAT) and mean annual precipitation (MAP) range from − 1.7 to 7.7 °C and 500–650 mm, respectively. For detailed information on soil and plant characteristics, see Table S3. Sampling took place from the north to the south along the transect in August and September of 2020. Five plants were chosen at random, and a 0.8 m wide × 0.8 m long× 1.0 m pit was excavated. Shaking the plants removed non-tightly adhering soil and acquired the entire root. Fine roots (root) and rhizosphere soil (soil) were collected, and five samples mixed into one sample in a disposable sterile bag. A total of 100 samples were collected from the 58 sites [30]. The rhizosphere soil and root samples were stored in refrigerators and immediately transported to the laboratory. The rhizosphere soil samples were sieved through a 2 mm mesh and thoroughly homogenized. The roots were sterilized by rinsing them in purified water for 10 min, followed by three rinses in sterile water. Then, they were sterilized for 2 min in 75% ethanol, followed by 3 rinses in sterile water. After that, a 5-minute sterilization in 0.5% sodium hypochlorite was performed, followed by three rinses in sterile water. Inoculating the medium with water from the final washing step of the root sterilization procedure did not result in any colony growth, indicating the effectiveness of the surface sterilization procedure [31]. Rhizosphere soil and root samples were stored at -80 °C before DNA extraction. All samples were identified by Professor Guoying Zhou (Northwest Institute of Plateau Biology, Chinese Academy of Science). The specimens were then stored at the herbarium in Northwest Institute of Plateau Biology, Chinese Academy of Science (No: QHGC-2010 ~ 2067).

### Soil physicochemical analyses and climate data acquisition

The soil total nitrogen (TN) was measured by a Vario EL (vario EL III CHNOS elemental analyzer, Elementar Analysensysteme GmbH). The soil total phosphorus (TP) and soil available phosphorus (AP) were determined using the molybdenum antimony phosphoric acid colorimetric method. Soil ammonium nitrogen (NH_4_^+^-N) and nitrate nitrogen (NO_3_^−^ N) were determined using an AQ1 discrete analyzer (SEAL Analytical, Inc., Mequon, WI, USA). The soil organic matter (SOM) was measured using the potassium dichromate oxidation method. The soil pH was measured in 1:2.5 soil suspensions in deionized water by pH meter. Data of mean annual temperature (MAT) and mean annual precipitation (MAP) at 30 arc seconds (~ 1km^2^) for each sampling site were obtained from the WorldClim database (https://worldclim.org/).

### Tropane alkaloid extraction and determination

In brief, 0.2 g dried powder samples were added with 10 mL 80% methanol (2% formic acid). The solution was sonicated for 30 min at room temperature, and then, the suspension was filtered through a 0.45 μm filter membrane for next analysis. The tropane alkaloids contents were determined by Agilent 1260 system (Agilent, USA). The samples were separated on an Atlantis T3 Column (250 mm×4.6 mm). Mobile phase A was acetonitrile, and mobile phase B was H_2_O with 0.1% trifluoroacetic acid. Further details were reported in previously published articles [4].

### DNA extraction and processing

Microbial DNA was extracted from the rhizosphere soil and root samples using FastDNA SPIN Kit according to the manufacturer’s instructions. Extracted DNA was quantified by NanoDrop 2000 UV-vis spectrophotometer (Thermo Scientific, Wilmington, USA) to determine the concentration. V5–V7 of bacterial 16 S rRNA (799F_1193R) and fungal ITS2 (ITS3F_ITS4R) were amplified by an ABI GeneAmp® 9700 PCR thermocycler (ABI, CA, USA) (Table S1). DNA samples were sequenced on an Illumina MiSeq PE300 platform (Illumina, San Diego, USA) at Shanghai Majorbio Bio-pharm Technology Co., Ltd. Raw sequences were joined using FLASH version 1.2.7 and quality filtered by fastp version 0.20. Operational taxonomic units (OTUs) were clustered at a 97% similarity level using UPARSE version 7.1. Taxonomic classification of OTUs was conducted using the SILVA database (SILVA 138, Max Planck institute, Bremen, Germany) for the 16 S rRNA gene and UNITE database 8.0 for the ITS region.

### Statistical analysis

All statistical analyses were conducted in the R program and visualized using the “ggplot2” package [32, 33]. Differences in the OTU richness between rhizosphere soil and root endosphere samples were tested using Student’s t-tests with the “stats” package. Differences in the relative abundance of dominant phylum between rhizosphere soil and root endosphere samples were tested using Wilcoxon’s rank-sum test with the “stats” package. Differences in the bacterial and fungal community composition between rhizosphere soil and root endosphere samples were performed by principal coordinates analysis (PCoA) based on Bray-Curtis distance and further tested by permutational multivariate analysis of variance (PERMANOVA) using the “vegan” package [34]. Beta nearest taxon index (βNTI) is used to quantify phylogenetic community structure. We calculated the beta Nearest Taxon Index (βNTI) using the “picante” package [35].

The distance–decay relationship (DDR) is one of the most common biogeographical patterns, in which the similarity of communities decreases with increasing distance [36]. At each sampling site, longitude and latitude were recorded by the Global Positioning System (GPS). The geographical distance between pairwise sampling sites was computed using the “geosphere” package [37]. Root-associated bacterial and fungal Bray–Curtis similarity was computed using the “vegan” package. Linear regression analysis was performed to assess the distance–decay relationships (DDRs) between the bacterial Bray–Curtis similarity and geographical distances. Mantel’s correlation was used to assess relationships between rhizosphere soil and root endosphere microbial community and environmental variables using the ‘linkET’ package [38].

Core OTUs of root-associated bacterial and fungal communities were selected as follows: (i) present in 70% rhizosphere soil and root endosphere samples; and (ii) with a relative abundance (RA) > 0.1%. Spearman correlation between genera abundance and anisodine, anisodamine, atropine, root nitrogen, and aboveground biomass was performed using the “psych” package [39]. Subsequently, we used random forest analysis to estimate the contributions of the genera abundance to the root anisodine content, aboveground biomass and nitrogen content using the “rfPermute” package with 9999 random permutations [40]. The percentage increases in the MSE (mean squared error) of variables were used to estimate the importance of genera abundance, and higher MSE% values imply more important variables.

We constructed co-occurrence networks at OTU level to investigate the species interactions of root bacterial communities. In order to rule out the influence of rare OTUs, the OTUs with more than 0.001% relative abundance were selected to calculate Spearman’s rank correlation coefficients. Spearman correlation coefficient r > |0.5| and *P* < 0.05 (FDR) were used for co-occurrence network construction with the “Hmisc” package [41]. The networks were visualized using Gephi platform (https://gephi.org/). The properties (average path length, network diameter, average degree, and average clustering coefficient) were determined to describe the network’s complexity. Degree, betweenness, and closeness centrality were evaluated for each node to detect topological properties at the node level. The difference in topological features between core root bacteria and others was tested using Wilcox test with the “stats” package.

## Results

### Root endophytes differ from the rhizosphere soil

**Fig. 1:**
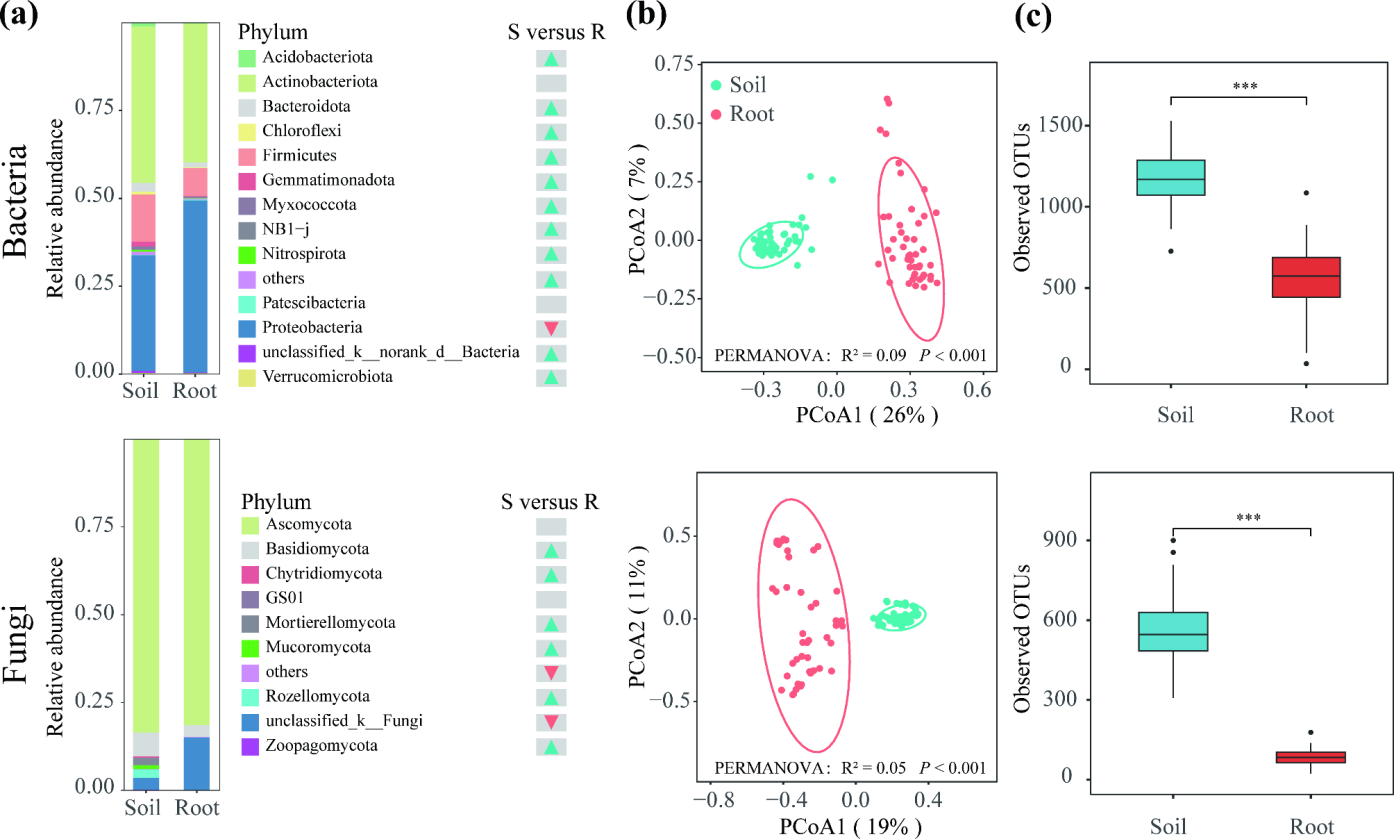
Microbial diversity and composition of *A.tanguticus*. (**a**) Microbial composition at phylum level (relative abundance of > 0.1%). Soil, rhizosphere soil; Root, root endosphere. Triangles depict statistically significant differences (Wilcoxon rank-sum test, *P* < 0.05). Red indicate enrichment in the root and blue indicate enrichment in the soil. (**b**) PCoA based on Bray–Curtis distance matrices depicting the distribution patterns of bacterial and fungal communities in rhizosphere soil and root endosphere. (**c**) Boxplots showing the OTU richness of bacterial and fungal communities in rhizosphere soil and root endosphere. Asterisks indicate significant differences between rhizosphere soil and root endosphere based on the Student’s t-test. The significance levels are as follows: *P* < 0.05, one asterisk (*); *P* < 0.01, two asterisks (**); *P* < 0.001, three asterisks (***)

The relative abundance of Proteobacteria was more abundant in the root endosphere than in the rhizosphere soil samples. (Wilcoxon rank-sum test, *P* < 0.001; Fig. 1a and Fig. S2). Contrariwise, the relative abundance of Firmicutes, Bacteroidota, Gemmatimonadota, Acidobacteriota, Myxococcota, unclassified_k__ norank_d__Bacteria, Nitrospirota, Verrucomicrobiota, NB1-j and Chloroflexi dropped in root endosphere compared to rhizosphere soil samples. The relative abundance of Unclassified_k_Fungi was more abundant in the root endosphere than in the rhizosphere soil samples (Wilcoxon rank-sum test, *P* < 0.001; Fig. 1a and Fig. S2). The relative abundance of Basidiomycota, Rozellomycota, Mortierellomycota, Mucoromycota, Chytridiomycota and Zoopagomycota dropped in root endosphere compared to rhizosphere soil samples. To explore the difference between the rhizosphere soil and the root endosphere microbiomes, we performed PCoA using a Bray–Curtis distance matrix. The PCoA revealed a distinct separation between rhizosphere soil and root endosphere microbiomes (Fig. 1b). PERMANOVA showed significant difference between the rhizosphere soil and the root endosphere (bacteria: R² = 0.09, *P* < 0.001; fungi: R² = 0.05, *P* < 0.001; Fig. 1b). Inspection of the alpha diversity showed a significantly reduced bacterial and fungal richness in the root microbiomes compared to that of the rhizosphere soil (Student’s t-test, *P* < 0.001; Fig. 1c). Overall, our findings showed a significant difference between rhizosphere soil and the root endosphere microbiomes, suggesting the importance of the host-filtering effect on root microbiota composition.

**Fig. 2:**
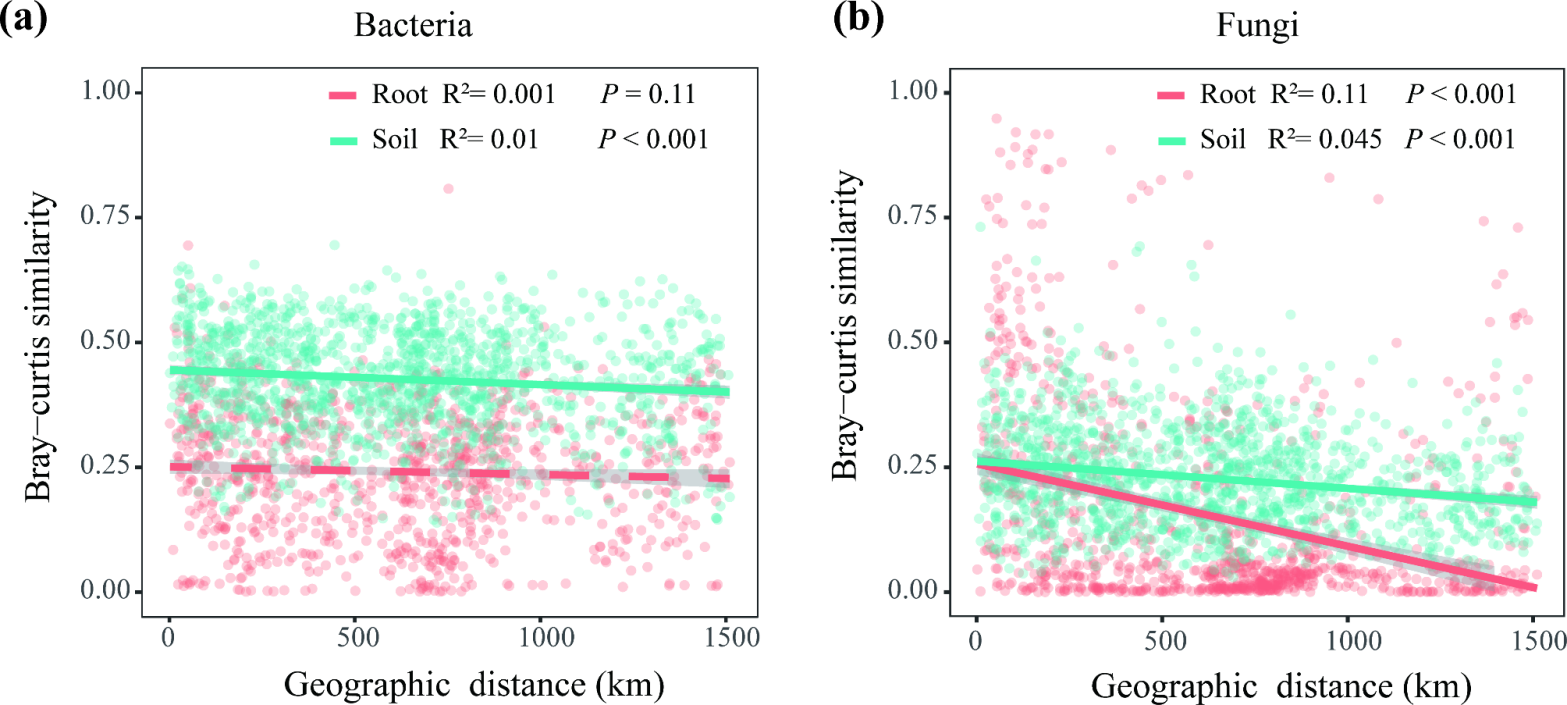
Distance–decay relationships between the bacterial (**a**) and fungal (**b**) community similarity (Bray–Curtis distances) and the geographical distances in rhizosphere soil and root endosphere habitats across the 58 sampling sites. The shaded region represents the 95% confidence limits of the regression estimates. The solid and dashed lines denote statistically significant (*P* < 0.05) and nonsignificant relationships, respectively. Soil, rhizosphere soil; Root, root endosphere

The distance-decay relationships (DDRs) for microbial communities in the rhizosphere soil and root endosphere were further analysed. We found a stronger relationship between geographic distance and fungal community similarity. The slopes of distance-decay curves were steeper for the root fungal communities (R^2^ = 0.11; *P* < 0.001; Fig. 2b) than rhizosphere soil fungal communities (R^2^ = 0.045; *P* < 0.001; Fig. 2b), indicating strong geographic structuring of the root fungi. However, we observed weak decay of rhizosphere soil bacterial community similarity with geographic distance (R^2^ = 0.01; *P* < 0.001; Fig. 2a), and no DDR was observed for root bacterial community (R^2^ = 0.001; *P* = 0.11; Fig. 2a). These findings show that the bacterial taxa that colonize roots were robust even though there were significant differences between the environmental conditions at various sites.

**Fig. 3:**
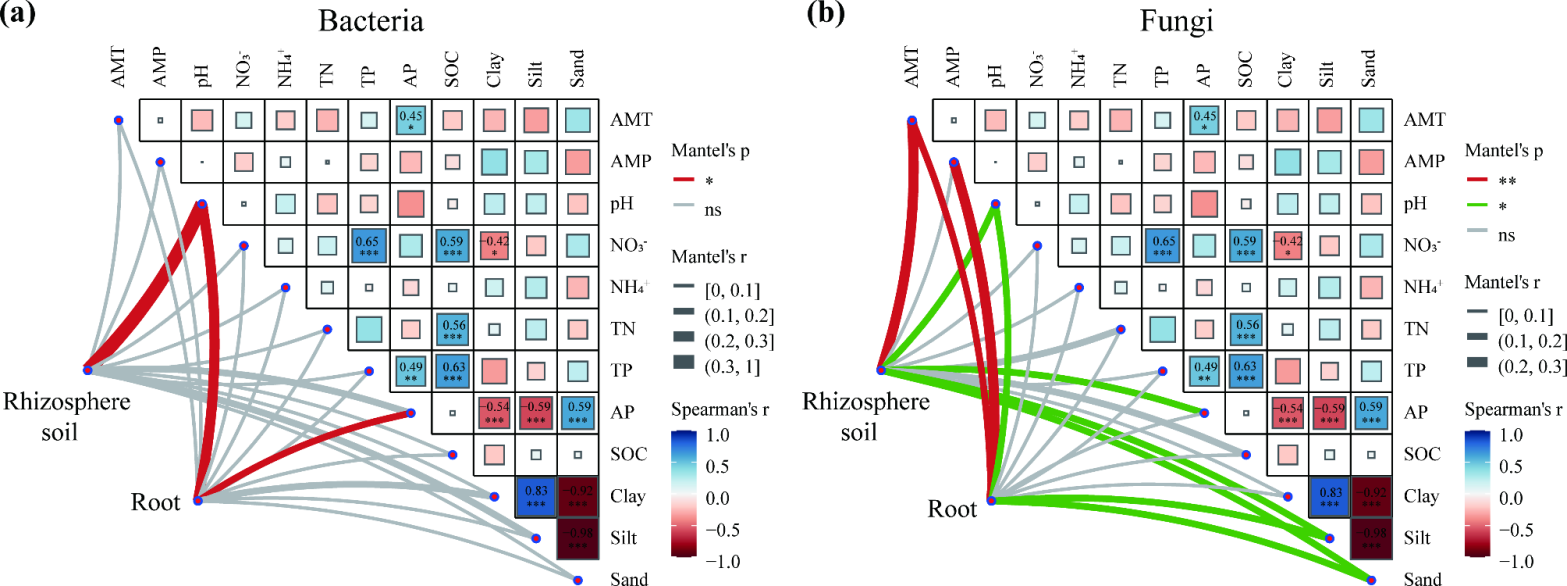
Mantel’s correlations between bacterial (**a**) and fungal (**b**) community compositions of rhizosphere soil and root endosphere, and soil environmental factors. The significance levels are as follows: *P* < 0.05, one asterisk (*); *P* < 0.01, two asterisks (**); *P* < 0.001, three asterisks (***). Coefficients without significance are not shown

Mantel’s correlation analysis revealed that pH was the only variable significantly associated with rhizosphere soil bacterial communities (Fig. 3a). Similarly, only pH and available phosphorus were found to be significantly related to root endosphere bacterial communities. Annual mean temperature, pH, available phosphorus, slit and sand were significantly related to rhizosphere soil fungal communities; Annual mean temperature, annual mean precipitation, pH, slit and sand were significantly related to root endosphere fungal communities (Fig. 3b). The above results indicate that root-related fungi are affected by more environmental factors, while bacteria are less affected by environmental factors, which further indicates that the root-related bacterial community of *A. tanguticus* was robust.

We calculated beta nearest taxon index (βNTI) values and compared the importance of stochastic and deterministic processes in the rhizosphere soil and root endosphere. The βNTI value between − 2 and 2 is a stochastic process, and if it is greater than 2 or less than − 2, it is a deterministic process [42]. The rhizosphere soil and root endosphere bacterial communities were dominated by deterministic processes, accounting for 73.65% and 56.52% respectively (Fig. S3). However, the rhizosphere soil and root endosphere fungal communities were dominated by stochastic processes, accounting for 78.48% and 59.81%, respectively.

### Identification root-inhabiting core microbiome

**Fig. 4:**
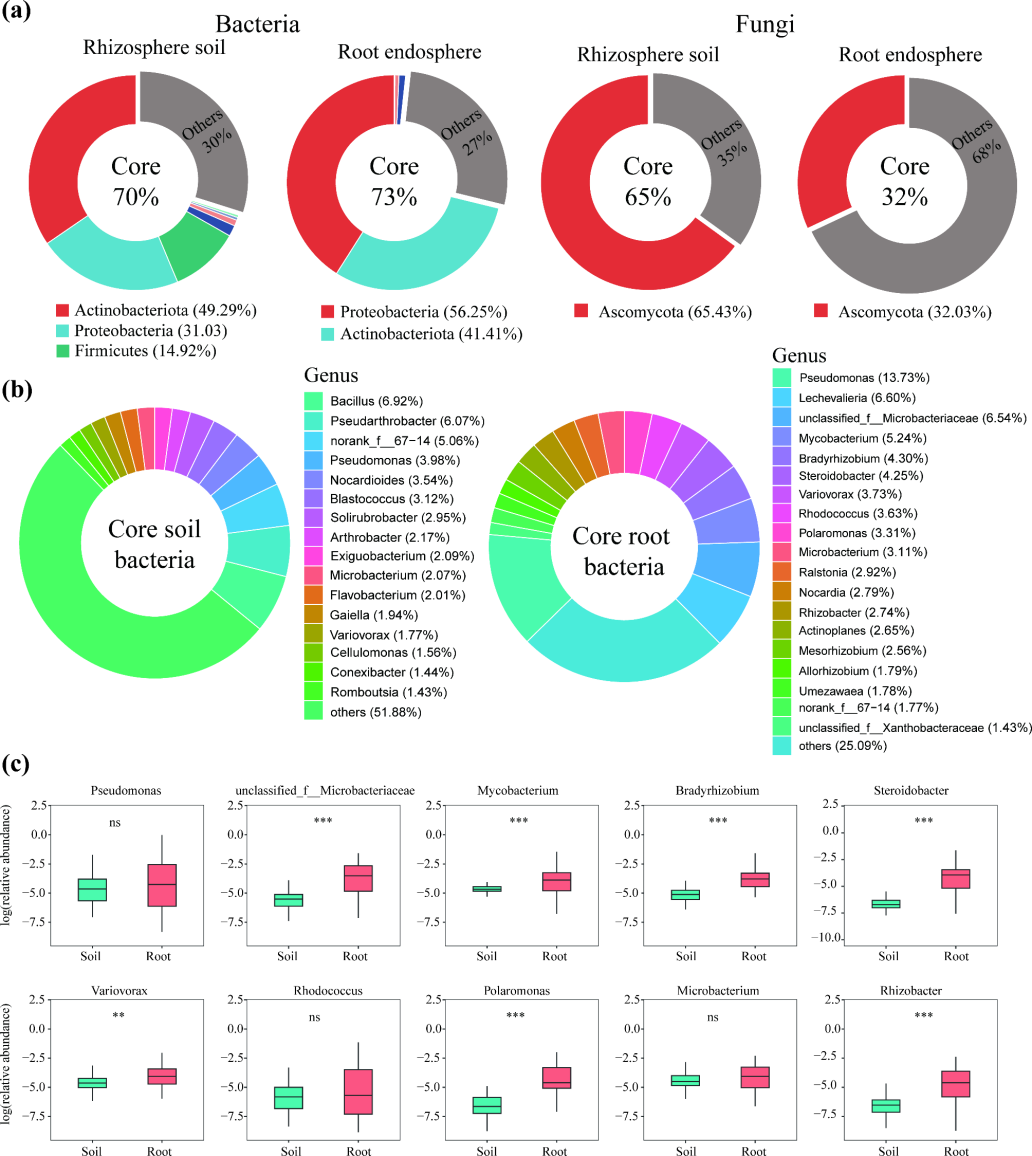
Core microbial composition of *A.tanguticus*. (**a**) Core microbial composition at phylum level. Numbers in parentheses indicate the proportion of relative abundance at phylum level. (**b**) Core bacterial composition at genus level. Numbers in parentheses indicate the proportion of relative abundance at genus level. (**c**) Differences in relative abundance between rhizosphere soil (soil) and root endosphere (root). Statistical significance was determined by the Wilcoxon rank-sum test. The significance levels are as follows: *P* < 0.05, one asterisk (*); *P* < 0.01, two asterisks (**); *P* < 0.001, three asterisks (***)

Taxa consistently found in a separate host-microbiome are defined as the host’s core microbiome. We selected OTU occurrence frequency greater than 0.7 and relative abundance greater than 0.1% in all samples as the core microbiome. A total of 218 OTUs out of 3578 OTUs and 102 OTUs out of 3412 OTUs were denoted as the core bacterial communities in the rhizosphere soil and root endosphere, respectively, accounting for 6.09% and 2.99% of all observed taxa. However, these taxa accounted for approximately 70% and 73% relative abundance of the total bacterial community in the rhizosphere soil and root endosphere, respectively (Fig. 4a). A total of 65 OTUs out of 4848 OTUs and 7 OTUs out of 1104 OTUs were denoted as the core fungal communities in the rhizosphere soil and root endosphere, respectively, accounting for 1.34% and 0.63% of all observed taxa. Similar to the findings for root-associated bacteria, the core rhizosphere soil fungal microbiome accounted for approximately 65% relative abundance of the total fungal community (Fig. 4a). However, we observed that the core root fungal microbiome comprised approximately 32% relative abundance of the total fungal community (Fig. 4a).

Although both the core root endosphere and rhizosphere soil bacteria were dominated by Proteobacteria and Actinobacteriota phylum, however, we further observed a significant difference in genus level between them. The core root bacteria genera were mainly comprised of *Pseudomonas* (13.73%), *Lechevalieria* (6.60%), *unclassified_f__Microbacteriaceae* (6.54%), *Mycobacterium* (5.24%), *Bradyrhizobium* (4.30%), *Steroidobacter* (4.25%), *Variovorax* (3.73%), *Rhodococcus* (3.63%), *Polaromonas* (3.31%), and *Microbacterium* (3.11%) (Fig. 4b). The core rhizosphere soil bacteria genera were mainly comprised of *Bacillus* (6.92%), *Pseudarthrobacter* (6.07%), *norank_f__67 − 14* (5.06%), *Pseudomonas* (3.98%), *Nocardioides* (3.54%), *Blastococcus* (3.12%), *Solirubrobacter* (2.95%), *Arthrobacter* (2.17%), *Exiguobacterium* (2.09%), and *Microbacterium* (2.07%) (Fig. 4b). The genera of *unclassified_f__Microbacteriaceae* (*P* < 0.001), *Mycobacterium* (*P* < 0.001), *Bradyrhizobium* (*P* < 0.001), *Steroidobacter* (*P* < 0.001), *Variovorax* (*P* < 0.01), *Polaromonas* (*P* < 0.001), and *Rhizobacter* (*P* < 0.001) are significantly enriched in root endosphere (Fig. 4c).

### Ecological roles of core root bacteria

**Fig. 5:**
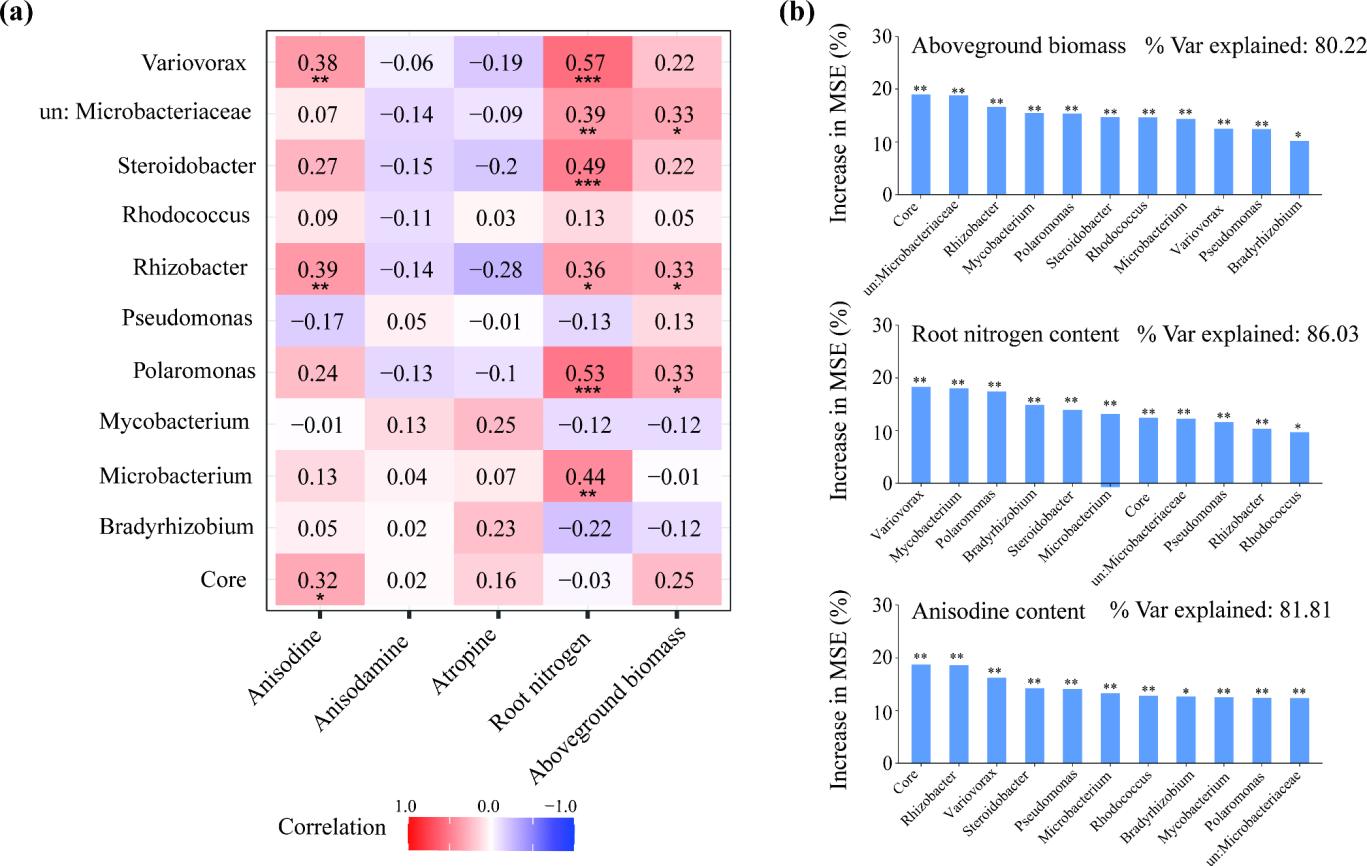
(**a**) Spearman correlation analysis of core genera (the top 10 abundance) and aboveground biomass, nitrogen content and root tropane alkaloids content of *Anisodus tanguticus*. The numbers in the graph indicate the R-value. The colors in the graph indicate the P-value. The red and blue indicate positive and negative correlations, and the color depth indicates strong correlation (* *P* < 0.05, ** *P* < 0.01, *** *P* < 0.01). (**b**) The predictions of core genera to aboveground biomass, nitrogen content and root anisodine content based on random forest regression analysis. “% var explained” means the proportion of variance explained. Asterisks indicate the factor that has a significant effect. The significance levels are as follows: *P* < 0.05, one asterisk (*); *P* < 0.01, two asterisks (**); *P* < 0.001, three asterisks (***)

We then explored the ecological roles of the core root bacteria in the physiological and ecological functions of plants. Based on relative abundance, we screened the top ten core bacterial genera. The relative abundances of some genera were significantly and positively correlated with anisodine content, such as *Variovorax*, *Rhizobacter*, and core bacteria (Fig. 5a). The results of random forest also show that core bacteria, *Rhizobacter*, and *Variovorax*, were the most important predictor for the root anisodine content (Fig. 5b). *Variovorax*, *unclassified_f__Microbacteriaceae*, *Steroidobacter*, *Rhizobacter*, *Polaromonas*, and *Microbacterium* are significantly positively correlated with root nitrogen content (Fig. 5a). The results of random forest show that *Variovorax*, *Mycobacterium*, and *Polaromonas* were the most important predictor for the root nitrogen content (Fig. 5b). *unclassified_f__Microbacteriaceae*, *Rhizobacter*, and *Polaromonas* are significantly positively correlated with aboveground biomass (Fig. 5a). The results of random forest show that core bacteria, *unclassified_f__Microbacteriaceae*, and *Rhizobacter*, were the most important predictor for the aboveground biomass (Fig. 5b). No genera related to anisodamine and atropine have been identified.

**Fig. 6:**
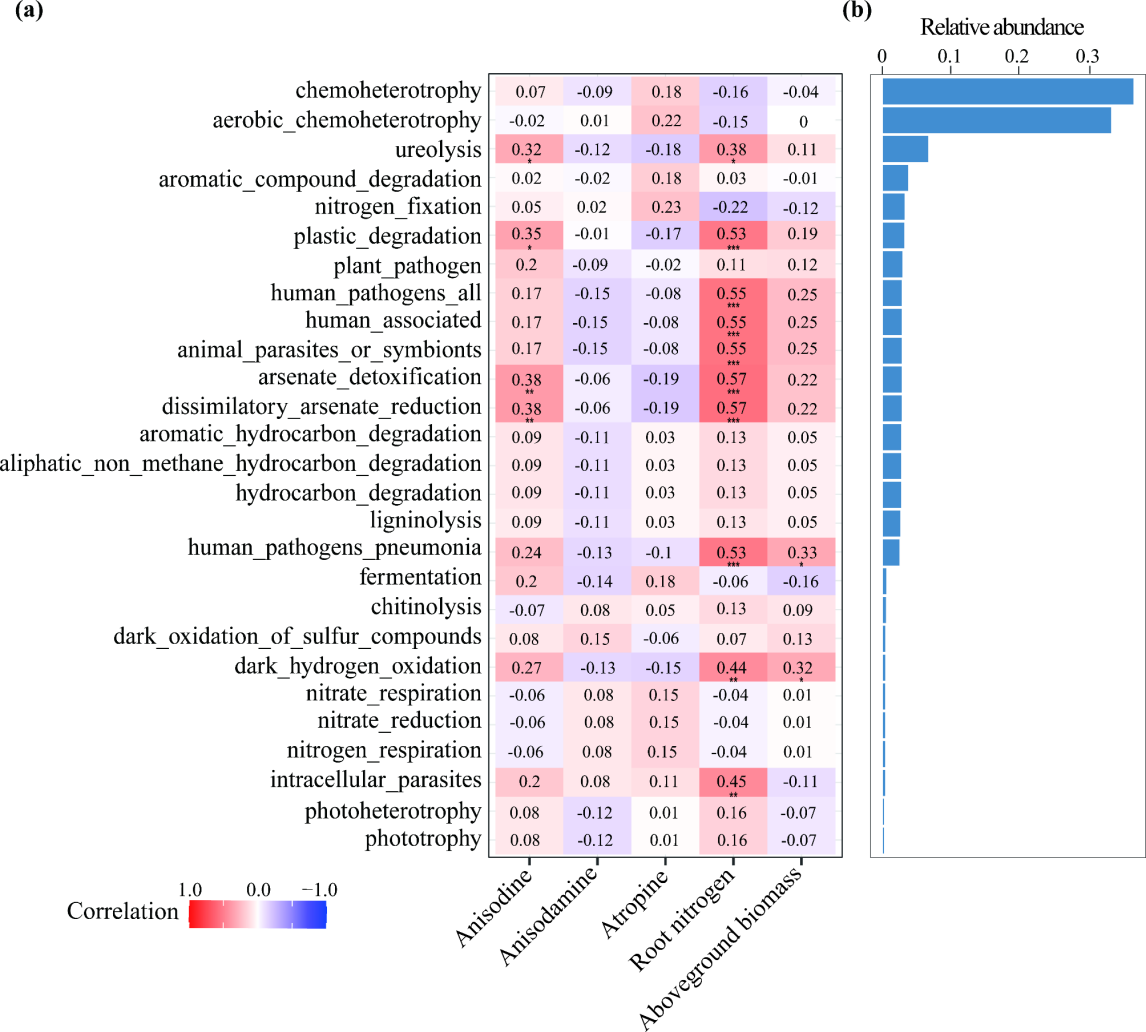
Core root bacterial functions annotated according to FAPROTAX. (**a**) Spearman correlation analysis of functional group and aboveground biomass, nitrogen content and root tropane alkaloids content of *Anisodus tanguticus*. The numbers in the graph indicate the R-value. The colors in the graph indicate the P-value. The red and blue indicate positive and negative correlations, and the color depth indicates strong correlation (* *P* < 0.05, ** *P* < 0.01, *** *P* < 0.01). (**b**) The relative abundance of the different functional groups

We further used FAPROTAX to functionally annotate root core bacteria. The dominant core root bacteria functions were mainly related to chemotrophy, aerobic_chemoheterotrophy, nitrogen cycling (ureolysis and nitrogen_fixation), pathogens (plant_pathogen, human_pathogens, animal_parasites, and intracellular_parasites), and material decomposition (aromatic_compound, plastic, hydrocarbon, aromatic_hydrocarbon, and aliphatic_non_methane_hydrocarbon) (Fig. 6b). Among them, the relative abundance of microorganisms related to nitrogen cycling (ureolysis), plastic_degradation, arsenate_detoxification, and dissimilatory_arsenate_reduction were significantly and positively correlated with anisodine content (Fig. 6a). The relative abundance of microorganisms related to nitrogen cycling (ureolysis), pathogens (human_pathogens, animal_parasites, and intracellular_parasites), plastic_degradation, arsenate_detoxification, and dissimilatory_arsenate_reduction were significantly and positively correlated with root nitrogen content (Fig. 6a). Only the relative abundance of microorganisms related to human_pathogens and dark_hydrogen_oxidation were associated with aboveground biomass (Fig. 6a). No functional group related to anisodamine and atropine have been identified. The above results suggest that nitrogen-cycling microorganisms and pathogenic bacteria play important roles in the plant growth, nitrogen and anisodine accumulation of *A. tanguticus*.

**Fig. 7:**
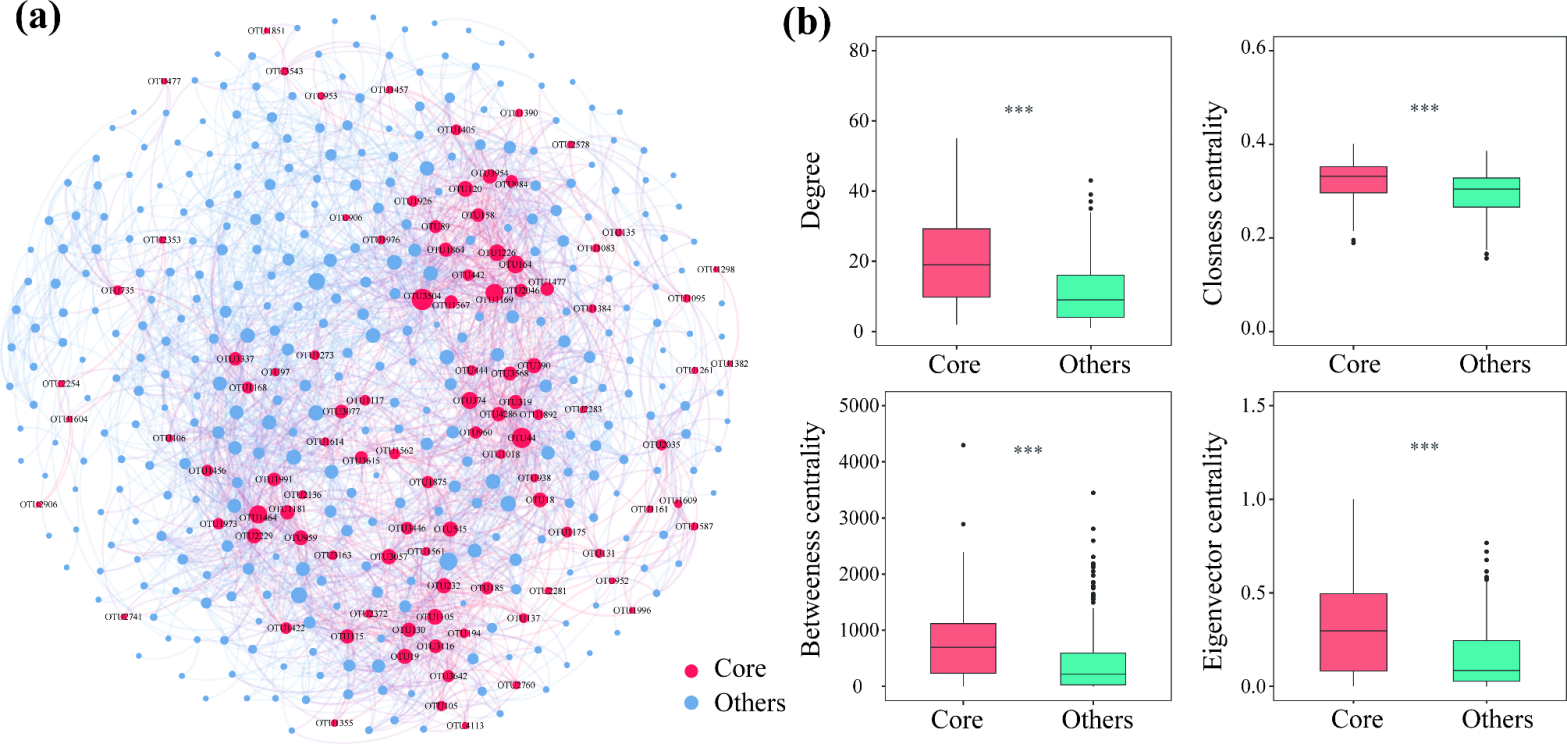
Co-occurrence patterns of the root bacterial community of *A.tanguticus*. (**a**) Co-occurrence networks for root bacterial community based on Spearman’s correlations (r > |0.5|, FDR-corrected *P* < 0.05). The red and blue dots represent core root microorganisms and others, respectively. (**b**) Node-level network topological features of different microbial taxa, specifically the degree, betweenness centrality, closeness centrality and eigenvector centrality. Statistical significance was determined by the Wilcoxon rank-sum test. The significance levels are as follows: *P* < 0.05, one asterisk (*); *P* < 0.01, two asterisks (**); *P* < 0.001, three asterisks (***)

Core microorganism with high connectivity is considered to have an important role in shaping microbial communities on plant hosts. Microbial network analysis has been frequently used to investigate the species interactions in complex microbial communities. We constructed core root bacterial co-occurrence networks based on correlation relationships (Fig. 7a). The network consisted of 440 nodes and 2921 edges (Table S2). Values of degree (*P* < 0.001), betweenness centrality (*P* < 0.001), eigenvector centrality (*P* < 0.001) and closeness centrality (*P* < 0.001) were significantly higher in core taxa than other taxa (Fig. 7b).

## Discussion

Our findings demonstrated that there were differences in the microorganisms present in the rhizosphere soil and the root endosphere. Previous research on *Zea mays*, *Triticum aestivum*, *Cycas panzhihuaensis* and *Populus* has consistently revealed that each plant compartment contains a different microbial assemblage [43–45]. Plants can choose microorganisms that are suitable to their ecological niche to inhabit distinct compartments, even across long distances and with varied fertilization treatments [43, 44]. These findings indicate that host plant selection is more important than environmental factors in shaping plant microbiome assembly. Furthermore, we discovered a substantial decrease in bacterial and fungal richness and diversity from soils to endophytes (Student’s t-test, *P* < 0.001; Fig. 1c). The host selection pressure gradually increased along the soil–plant continuum [44]. There is a significant loss of microbial diversity from soil to plant tissues, and only a few number of bacteria may colonize and survive in root tissues, owing to the following causes. On the one hand, microbes need to pass the host immune system’s filter, and on the other hand, microbes need to adapt to the microenvironment of plant tissue [43].

Host selection often results in a robust core of microbial taxa in plant roots, independent of geographic distance. It has been discovered that the stem xylem of maize plants in China’s primary agricultural production zones selectively recruits highly conserved bacterial microbiota [27]. Studies on *Arabidopsis thaliana* across European sites suggested convergence in root bacterial microbiota composition [46]. In our study, we also observed that the root of *A. tanguticus* recruited a core bacterial microbiota independent of geographic distances. A total of 102 OTUs were identified as the core bacterial communities in the root endosphere. Although comprising only 2.99% of all observed taxa, these microorganisms accounted for approximately 73% relative abundance of the total bacterial community in the root endosphere (Fig. 3a). Root core bacteria are mainly composed of genera such as *Pseudomonas*, *Lechevalieria*, *unclassified_f__Microbacteriaceae*, *Mycobacterium*, *Bradyrhizobium*, *Steroidobacter*, *Variovorax*, *Rhodococcus*, *Polaromonas*, and *Microbacterium*. The root core bacteria of *Arabidopsis thaliana* across European are mainly composed of *Pseudomonas*, *Burkholderia*, *Bradhyrhizobium*, *Polaromonas*, *Ralstonia*, and *Massilia* [46]. The *Bradyrhizobium*, *Rhizobium*, *Burkholderia*, and *Azospirillum* genera have been identified as a core root microbiome across multiple plant phyla in a soil chronosequence in Australia [47]. The *Pseudomonas*, *Promicromonospora*, *Massilia*, *Umezawaea*, *Lechevalieria*, *Devosia*, *Duganella* and *Streptomyces* genera have been identified as a core root microbiome across ninety-five winter wheat varieties [26]. Despite significant phylogenetic diversity among these plant species, similar microbial compositions suggest that plant root microbial composition may be evolutionarily conserved at global scales. A core root bacterial community was established before the evolution of modern plant lineages, and root-associated bacterial communities evolved along with their plant hosts [47].

*Pseudomonas* genera was the most dominant in the roots of *Arabidopsis thaliana*, wheat, and *Anisodus tanguticus*, pointing to *Pseudomonas* taxa as robust root colonizers. Although *Pseudomonas* is a potential pathogen, it can utilise a wide niche breadth to suppress other pathogens through resource competition. For example, high *Pseudomonas* diversity reduced the pathogen *Ralstonia solanacearum* density in the tomato rhizosphere microbiome [48]. *Bradhyrhizobium*, *Rhizobacter* and *Rhizobium* genera are known to fix nitrogen for legumes [49]. In this study, *Bradhyrhizobium* and *Rhizobacter* were significantly enriched in the roots of *A. tanguticus*, indicating their close association with non-leguminous plants as well. Moreover, studies have shown that the molecular mechanisms underlying their colonization in non-leguminous plants such as *Arabidopsis*, corn, and tomato are similar [50]. The *Steroidobacter* genera also play a crucial role in the plant-soil nitrogen cycle [51]. *Variovora* genera can regulate the concentration of growth hormone to maintain normal root development through interaction with plants [52]. *Polaromonas* are commonly found in polar and alpine microbial communities worldwide [53].

Further studies showed that *Rhizobacter*, *Variovora*, *Polaromonas*, *Steroidobacter*, *unclassified_f__Microbacteriaceae*, *Microbacterium*, and *Mycobacterium* were significantly positively correlated with root anisodine content, root nitrogen content and aboveground biomass of *A. tanguticus*. *Rhizobacter*, *Variovora*, and *Steroidobacter* can promote nitrogen uptake by plants through biological nitrogen fixation, maintaining normal root development and participating in the nitrogen cycle. This also explains why they are significantly positively correlated with root nitrogen content and aboveground biomass of *A. tanguticus*. Anisodine are a class of naturally occurring organic nitrogen-containing chemicals found primarily in plants [54]. Research shows that nitrogen supplementation increases the synthesis of anisodine. *unclassified_f__Microbacteriaceae*, *Microbacterium*, and *Mycobacterium* are mostly pathogenic bacteria [55]. The results of FAPROTAX functional annotation and spearman correlation analysis also indicate that nitrogen-cycling microorganisms and pathogenic bacteria play important roles in the plant growth, nitrogen and anisodine accumulation of *A. tanguticus*. This may be related to the living habits of *A. tanguticus*, which usually likes to live near herdsmen’s sheep pens and cattle pens. Therefore, its roots are colonized by a large number of animal and human pathogenic bacteria. However, how they interact with and promote the growth of *A. tanguticus* remains to be studied further.

The relationship between core microorganisms and their host is persistent. Therefore, it is crucial to understand the ecological roles of the core bacteria to comprehend the assembly and stability of root endophytic bacteria. In network analysis, topological features with high values indicate that a node holds a central and core position within the network. Higher topological eigenvalues for core taxa were found in our investigation, indicating the presence of core microorganisms at the network’s core (Fig. 7). This may be due to the fact that the core microbiota occupies a wider ecological niches [56]. The core microorganisms are able to establish a stable relationship with their hosts regardless of environmental variation. The colonization of many other microorganisms is influenced by the colonization of core bacteria. Even pathogens that are harmful for plants’ health could operate as crucial hubs that reduce bacterial diversity and increase the relative abundance of other microbes [57].

## Conclusions

Despite sampling at a distance of 1500 km, the root of *A. tanguticus* selectively recruits a core group of bacteria that is dominated by a small number of taxa. The composition of this core microbial taxon is similar to the composition found in a variety of phylogenetically distant plants such as *Arabidopsis* and wheat. This suggests that the microbial composition of plant roots may be evolutionarily conserved on a global scale. Our further analyses revealed that the core root bacteria were significantly and positively correlated with root anisodine contents, aboveground biomass, and root nitrogen contents of *A. tanguticus*. Among these, nitrogen cycle taxa and pathogenic bacteria play important roles. This research enhances our understanding of the interactions between microorganisms and plants, and may contribute to the development of strategies to manage root microbiota for sustainable production of herbal medicines.

### Electronic supplementary material

Below is the link to the electronic supplementary material.

**Additional file 1**: Fig. S1. Map showing sampling sites in the Qinghai-Tibetan Plateau. Fig. S2. Comparison of relative abundance between rhizosphere soil (soil) and root endosphere (root) samples for bacteria and fungi at phyla level. Relative abundance measured in soil and root samples across the 58 *A.tanguticus* populations was aggregated at the phyla level. The differences between compartments were determined using Wilcoxon rank-sum test. The asterisk (*) indicates significant differences. Fig. S3. Boxplots showing the βNTI values in bacterial (a) and fungi (b) community. Statistical significance was determined by the Wilcoxon rank-sum test. The dotted line indicates − 2 and 2. The upper number represents a deterministic process, and the lower number represents a stochastic process. The significance levels are as follows: *P* < 0.05, one asterisk (*); *P* < 0.01, two asterisks (**); *P* < 0.001, three asterisks (***). Soil, rhizosphere soil; Root, root endosphere. Table S1. Primers utilized in this study to profile bacterial and fungal communities in soil and root samples. Table S2. Root bacterial network topological parameters

**Additional file 2**: Table S3. Summary of the geospatial, climate, soil and plant characteristics of the sampling sites

## Data Availability

All data generated or analysed during this study have been submitted to the NCBI Sequence Read Archive (SRA) database under the accession numbers PRJNA837402 (16 S) and PRJNA837692 (ITS) and its supplementary information files. The datasets used and/or analysed during the current study are available from the corresponding author on reasonable request.
