# Supplementary material for: A core root bacteria contribute to plant growth and anisodine accumulation of Anisodus tanguticus
Source: BMC Plant Biol. 2023 Dec 19;23:655. doi: 10.1186/s12870-023-04690-1 (PMC10729362; doi:10.1186/s12870-023-04690-1)
Supplement: Supplementary file 1 — Additional file 1: Fig. S1. Map showing sampling sites in the Qinghai-Tibetan Plateau. Fig. S2. Comparison of relative abundance between rhizosphere soil (soil) and root endosphere (root) samples for bacteria and fungi at phyla level. Relative abundance measured in soil and root samples across the 58 A.tanguticus populations was aggregated at the phyla level. The differences between compartments were determined using Wilcoxon rank-sum test. The asterisk (*) indicates significant differences. Fig. S3. Boxplots showing the βNTI values in bacterial (a) and fungi (b) community. Statistical significance was determined by the Wilcoxon rank-sum test. The dotted line indicates − 2 and 2. The upper number represents a deterministic process, and the lower number represents a stochastic process. The significance levels are as follows: P < 0.05, one asterisk (*); P < 0.01, two asterisks (**); P < 0.001, three asterisks (***). Soil, rhizosphere soil; Root, root endosphere. Table S1. Primers utilized in this study to profile bacterial and fungal communities in soil and root samples. Table S2. Root bacterial network topological parameters [file 12870_2023_4690_MOESM1_ESM.docx]

**A** **core root bacteria** **contribute to plant growth and anisodine accumulation of *Anisodus tanguticus***

Bo Wang^1,3^, Chen Chen^2^, Yuanming Xiao^1^, Kaiyang Chen^1,3^, Juan Wang^5^, Lingling Wang^4^, Jianan Li^1,3^, Zongxiu Kang^6^, and Guoying Zhou^1,*^

1 CAS Key Laboratory of Tibetan Medicine Research, Northwest Institute of Plateau Biology, Xining 810008, China

2 College of Life Sciences, Huaibei Normal University, Huaibei, China

3 University of Chinese Academy of Sciences, Beijing 100049, China

4 Resource institute for Chinese and Ethnic Materia Medica, Guizhou University of Traditional Chinese Medicine, Guiyang 550000, China

5 Qinghai University, Xining 810016, China

6 Datong Beichuan Heyuan District National Nature Reserve, Xining 810100, China

*Corresponding author: Dr. Guoying Zhou, Tel: +86-971-6159630, Fax: +86-971-

6143282, E-mail: zhougy@nwipb.cas.cn

ORCID: https://orcid.org/0000-0003-2485-6172

Address: 23# Xinning Road, Xining, Qinghai, P. R. China 810008

Table S1 Primers utilized in this study to profile bacterial and fungal communities in soil and root samples

| **Name** | **Bacteria 16s V5/V7** |
| --- | --- |
| 799F | AACMGGATTAGATACCCKG |
| 1193R | ACGTCATCCCCACCTTCC |
|  | **Fungi ITS2** |
| ITS3F | GCATCGATGAAGAACGCAGC |
| ITS4R | TCCTCCGCTTATTGATATGC |

Table S2 Root bacterial network topological parameters

| Topological parameters | Value |
| --- | --- |
| Number of nodes | 440 |
| Number of edges | 2921 |
| Average degree | 13.277 |
| Diameter | 10 |
| Average path length | 3.423 |
| Average Clustering Coefficient | 0.387 |
| Modularity | 0.543 |


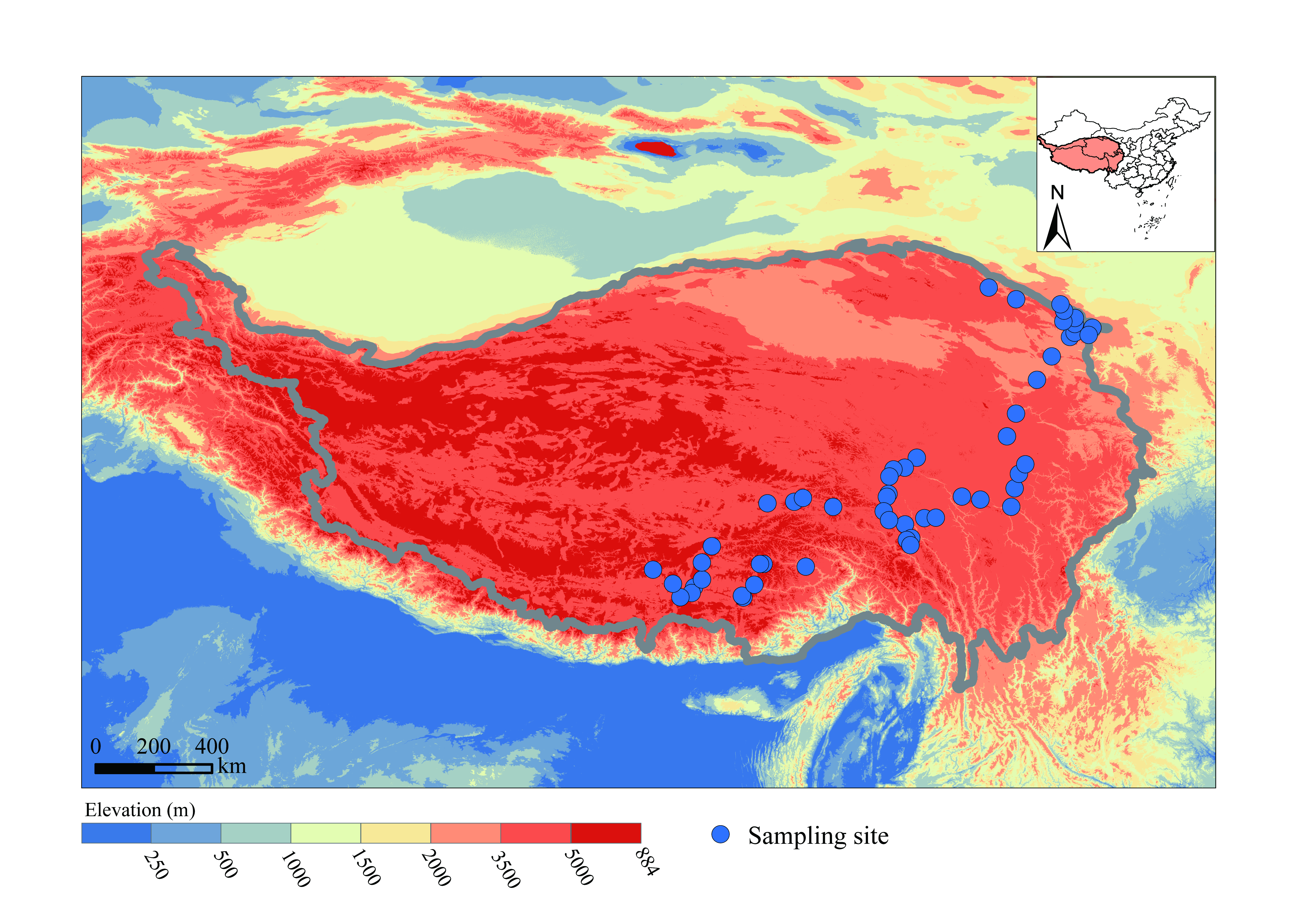


Fig. S1 Map showing sampling sites in the Qinghai-Tibetan Plateau





Fig. S2 Comparison of relative abundance between rhizosphere soil (soil) and root endosphere (root) samples for bacteria and fungi at phyla level. Relative abundance measured in soil and root samples across the 58 *A.tanguticus* populations was aggregated at the phyla level. The differences between compartments were determined using Wilcoxon rank-sum test. The asterisk (*) indicates significant differences.


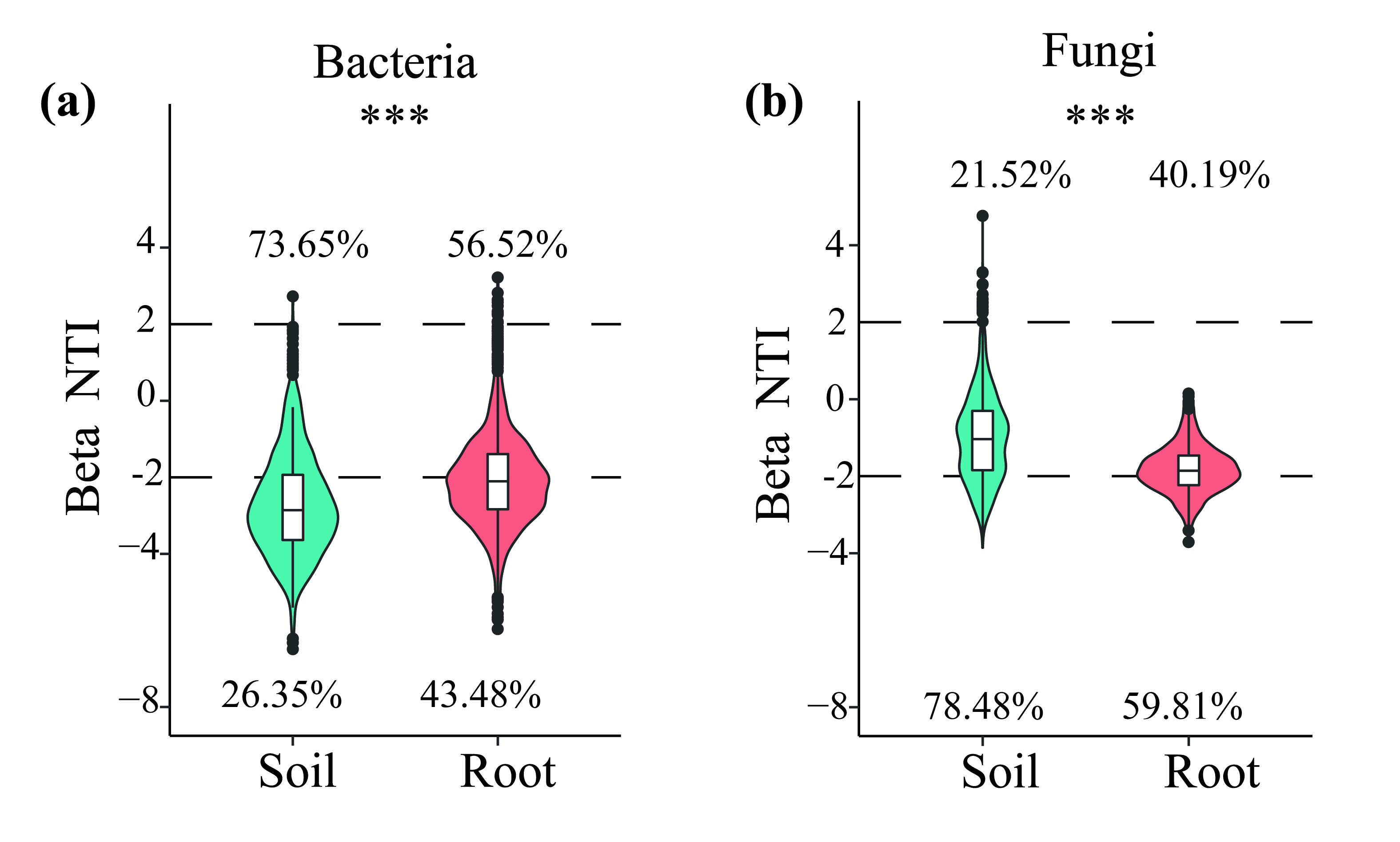


Fig. S3 Boxplots showing the βNTI values in bacterial (a) and fungi (b) community. Statistical significance was determined by the Wilcoxon rank-sum test. The dotted line indicates -2 and 2. The upper number represents a deterministic process, and the lower number represents a stochastic process. The significance levels are as follows: *P* < 0.05, one asterisk (*); *P* < 0.01, two asterisks (**); *P* < 0.001, three asterisks (***). Soil, rhizosphere soil; Root, root endosphere.
